# Differences between males and females in infectious diseases notifications in the EU/EEA, 2012 to 2021

**DOI:** 10.2807/1560-7917.ES.2024.29.33.2300655

**Published:** 2024-08-15

**Authors:** Julien Beauté, Francesco Innocenti

**Affiliations:** 1 European Centre for Disease Prevention and Control (ECDC), Stockholm, Sweden; 2 Epidemiology Unit, Regional Health Agency of Tuscany, Florence, Italy

**Keywords:** infectious disease, surveillance, sex difference, gender difference, Europe

## Abstract

**Background:**

There are differences between males and females for most diseases both for exposure and course of illness, including outcome. These differences can be related to biological sex or gender i.e. socio-cultural factors that may impact exposure and healthcare access.

**Aim:**

We aimed to quantify differences between males and females in infectious disease notifications in Europe and identify countries with these differences significantly different from the European Union and European Economic Area (EU/EEA) average.

**Methods:**

Notifiable infectious disease surveillance data are reported by EU/EEA countries to ECDC. We retrieved surveillance data for 2012−2021. Using a cut-off median of annual disability-adjusted life years above 1 per 100,000 population, we included 16 infectious diseases. We calculated median male proportion and interquartile range by disease, year, country and age group and used boxplots to identify outliers.

**Results:**

For campylobacteriosis, acute hepatitis B, Legionnaires’ disease, malaria and HIV and AIDS, all countries had male proportion above 50%. Most countries had a male proportion below 50% for pertussis (25/28 countries), STEC infection (21/28 countries) and *Chlamydia trachomatis *infection (16/24 countries). *Chlamydia trachomatis* infection and listeriosis showed the greatest dispersion of male proportion across age groups. Most outliers were countries reporting few cases.

**Conclusion:**

We observed important differences in male proportion across infectious disease notifications in EU/EEA countries. For some diseases with high male proportions in all countries, such as HIV and hepatitis B, behaviours play a role in disease transmission. Screening offered to specific populations may explain differences across countries for example for *C. trachomatis* infection.

**Key public health message**

**What did you want to address in this study and why?**

There are differences between males and females for most diseases both for exposure and course of illness, including outcome. Using surveillance data, we aimed to quantify sex differences in infectious disease notifications in Europe and possibly identify countries with sex differences significantly higher/smaller than the EU/EEA average to help us understand possible drivers for these differences.

**What have we learnt from this study?**

There are important differences in the proportion of male cases for some infectious disease notifications in EU/EEA countries such as *C. trachomatis* infection, HIV or malaria. However, between country variations were relatively small suggesting there may be common drivers for differences across countries. Country-specific screening policies may explain differences observed in some countries standing out from the EU/EEA average.

**What are the implications of your findings for public health?**

Our findings highlight the importance of documenting characteristics of surveillance systems likely to impact differences between males and females, such as screening policies. It is also important to collect case information possibly associated with sex/gender difference, such as occupation or transmission mode.

## Introduction

Differences between males and females for most diseases have been reported both in terms of exposure and course of illness, including disease outcome (e.g. death or recovery) [1]. These differences can be biological sex-related, i.e. due to anatomical or hormonal differences, or gender-related, i.e. associated with socio-cultural factors that may impact behaviours both for exposure e.g. women being more likely than men to be caregivers for the sick for both formal and informal care and healthcare access. These differences can change over the life cycle (e.g. conditions associated with menopause) and over time (e.g. changing gender differences in occupational choices). Although most of the leading causes of disability-adjusted life years (DALYs) identified by the Global Burden of Diseases, Injuries, and Risk Factors Study were similar for both sexes, some conditions, such as liver cirrhosis or lung cancer, were leading causes in males only, while others, such as low back pain, were leading causes in females only [2]. It is difficult to disentangle biological sex-related from gender-related differences. However, there are suggested examples of biological sex differences such as immune responses linked to COVID-19 outcomes [3] or sex hormones playing a role in cardiovascular disease [4]. Other studies pointed out biological sex differences in healthcare access, such as for tuberculosis (TB) for which males are disadvantaged [5].

In a report addressing sex and gender in epidemic-prone diseases, the World Health Organization (WHO) concluded that systematic evidence on sex and gender difference was scarce although there were strong indications that sex and gender played an important role in transmission and control [1]. The authors recommended routine collection of information on sex, occupational status and pregnancy in surveillance data. The European Centre for Disease Prevention and Control (ECDC) processes surveillance data on ca 60 infectious diseases and related health issues from 30 European Union and European Economic Area (EU/EEA) countries. Although information on sex is systematically collected, information on occupation or pregnancy status is only collected when associated with a specific risk, such as the risk of severe pregnancy outcome associated with Zika virus infection [6]. Many infectious diseases are more common in males e.g. Legionnaires’ disease where male-to-female rate ratio is 2.6:1 [7], whereas some, such as *C. trachomatis* infections, have a male-to-female ratio below one, 0.9 in 2022 in Europe [8]. This ratio may vary across age groups, as with for Legionnaires’ disease where it increases with age from 1.5:1 below 20 years to 3.3 in those aged 40–49 years [7]. Surveillance data also suggest sex differences in setting of infection and disease outcome. The odds of having healthcare-associated Legionnaires’ disease are higher for women, but females are less likely to die than males [9].

The objectives of this study were (i) to quantify sex differences in infectious disease notifications in Europe; and (ii) to identify countries with sex differences significantly higher/smaller than the EU/EEA average to help us understand possible drivers for these differences.

## Methods

### Data sources

We conducted a retrospective analysis of surveillance data. We retrieved 10 years (2012–2021) of case-based data on 15 March 2023 from The European Surveillance System (TESSy) for infectious diseases notifiable at EU level with the highest burden measured in DALYs according to the Burden of Communicable Diseases in Europe study [10]. Using the arbitrary cut-off median of annual DALYs above 1 per 100,000 population during 2009–2013 to ensure sufficient data for all diseases, 16 infectious diseases were included. We did not include seasonal influenza because it is not reported in a case-based format, or COVID-19 for which data collection started in 2020. Since EU case definitions do not always require a laboratory test to ascertain probable cases, we decided to include cases as per ECDC routine surveillance outputs. Thus, we included all cases meeting the EU case definition (confirmed and probable) for HIV/AIDS, Legionnaires’ disease and TB [11], but we only included confirmed cases of campylobacteriosis, *C. trachomatis* infection, invasive *Haemophilus influenzae* disease (IHID), acute and chronic hepatitis B, listeriosis, malaria, invasive meningococcal disease (IMD), pertussis, invasive pneumococcal disease (IPD), salmonellosis and Shiga toxin-producing *Escherichia coli* (STEC) infection. Detailed information on surveillance systems at country level for each disease are available elsewhere [12].

### Data analysis

We excluded cases reported with sex recorded as ‘other’, i.e. neither male nor female or missing information. We computed male proportion by disease, country, year and by six age groups (< 5, 5–14, 15–24, 25–44, 45–64, and ≥ 65 years) for cases. We compared male and female proportions using a z-test, assuming a normal distribution. We preferred male proportion over male-to-female ratio because it allowed us to have values even when there were no females. For each disease, we computed median, range and interquartile range (IQR) of country proportions of males. We defined outliers as countries with proportions of males beyond lower and upper adjacent values (i.e. below first quartile (Q1): Q1 - 1.5 × IQR, or above third quartile (Q3): Q3 + 1.5 × IQR) [13]. We ran linear regressions to assess trends over time. For trend analyses, we only included countries with no gap in the timeseries. We did not run trend analyses for diseases for which the countries with no gap in the timeseries accounted for less than 80% of the cases reported over the study period. All statistical tests were performed with a significance level of less than 0.05.

## Results

### Case overview

We included 5,656,031 cases reported by 30 EU/EEA countries during 2012–2021 (we excluded United Kingdom data as it left the EU in January 2020), of which 2,994,557 (52.9%) were males and 2,661,474 (47.1%) females (Table 1). We excluded 370 cases reported as other sex.

**Table 1:** Number and proportion of all cases by sex for 16 infectious diseases, by disease, 30 EU/EEA countries, 2012–2021

Disease	Males			Females			Total	Countries
	n	%	95% CI	n	%	95% CI	n	n
AIDS	119,721	74.2	74.0–74.4	41,558	25.8	25.6–26.0	161,279	30
Campylobacteriosis	840,131	53.6	53.5–53.7	726,544	46.4	46.3–46.5	1,566,675	28
*Chlamydia trachomatis* infection	688,943	43.1	43.0–43.2	910,327	56.9	56.8–57.0	1,599,270	24
Hepatitis B (acute)	15,442	69.3	68.7–69.9	6,834	30.7	30.1–31.3	22,276	27
Hepatitis B (chronic)	40,565	58.4	58.1–58.8	28,866	41.6	41.2–41.9	69,431	24
HIV	209,192	76.6	76.5–76.8	63,758	23.4	23.2–23.5	272,950	30
Invasive *Haemophilus influenzae* disease^a^	11,504	49.7	49.1–50.4	11,634	50.3	49.6–50.9	23,138	30
Invasive meningococcal disease	10,638	51.0	50.3–51.7	10,224	49.0	48.3–49.7	20,862	27
Invasive pneumococcal disease	79,950	55.9	55.6–56.2	63,092	44.1	43.8–44.4	143,042	27
Legionnaires’ disease	56,462	70.4	70.1–70.7	23,712	29.6	29.3–29.9	80,174	30
Listeriosis	11,556	54.7	54.0–55.3	9,579	45.3	44.7–46.0	21,135	28
Malaria	36,385	66.2	65.8–66.6	18,609	33.8	33.4–34.2	54,994	28
Pertussis	106,068	43.9	43.7–44.1	135,754	56.1	55.9–56.3	241,822	28
Salmonellosis	413,746	50.2	50.0–50.3	411,175	49.8	49.7–50.0	824,921	29
STEC infection	27,844	45.6	45.2–46.0	33,195	54.4	54.0–54.8	61,039	28
Tuberculosis	326,410	66.2	66.1–66.3	166,613	33.8	33.7–33.9	493,023	30
**Total**	**2,994,557**	**52.9**	**NA**	**2,661,474**	**47.1**	**NA**	**5,656,031**	**30**

NA: not applicable; STEC: Shiga toxin-producing *Escherichia coli.*

^a^ Distributions were statistically significant between males and females at the 5% level for all diseases except Invasive *Haemophilus influenzae* disease.

**Figure 1:**
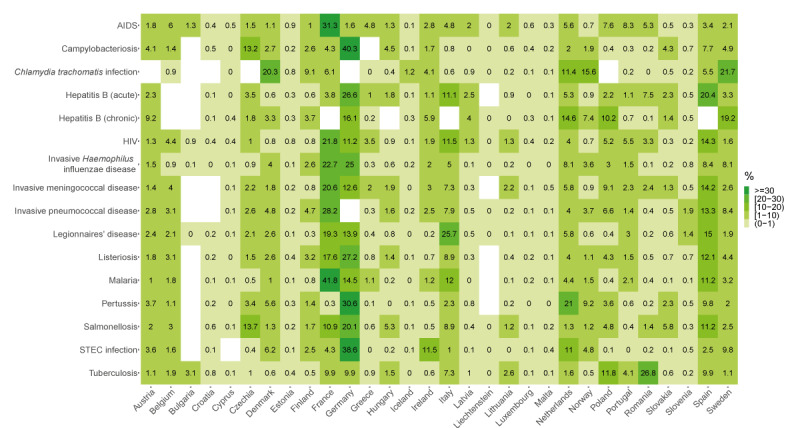
Proportion of cases reported by country for 16 infectious diseases, 30 EU/EEA countries, 2012–2021

The number of reporting countries ranged from 24 (*C. trachomatis* infection and chronic hepatitis B) to 30 (HIV/AIDS, IHID, Legionnaires’ disease and TB). Of the 30 EU/EEA countries, 25 reported data for at least 15 of the 16 diseases included in the analysis. Belgium and Germany reported data for 14 diseases, Croatia for 13 diseases, Liechtenstein for 11 diseases and Bulgaria for five diseases (Figure 1). Three diseases (*C. trachomatis* infection, campylobacteriosis and salmonellosis) accounted for 70.6% of all reported cases. Five countries (Germany, France, Sweden, Spain and Denmark) accounted for 51.7% of all reported cases (Table 2).

STEC: Shiga toxin-producing *Escherichia coli.*

**Table 2:** Number and proportion of all cases by sex for 16 infectious diseases, by country, 30 EU/EEA countries, 2012–2021

Country	Males			Females			Total
	n	%	95% CI	n	%	95% CI	n
Austria	65,771	55.9	55.6–56.2	51,872	44.1	43.8–44.4	117,643
Belgium	57,136	53.7	53.4–54.0	49,352	46.3	46.0–46.6	106,488
Bulgaria	13,961	70.9	70.3–71.6	5,720	29.1	28.4–29.7	19,681
Croatia	11,262	58.2	57.5–58.9	8,099	41.8	41.1–42.5	19,361
Cyprus	2,704	68.9	67.4–70.3	1,222	31.1	29.7–32.6	3,926
Czechia	179,452	51.8	51.6–51.9	167,236	48.2	48.1–48.4	346,688
Denmark	177,871	42.9	42.8–43.1	236,628	57.1	56.9–57.2	414,499
Estonia	8,708	35.1	34.5–35.7	16,108	64.9	64.3–65.5	24,816
Finland	102,073	45.8	45.6–46.0	120,998	54.2	54.0–54.4	223,071
France	280,082	54.9	54.7–55.0	230,378	45.1	45.0–45.3	510,460
Germany	551,033	53.6	53.5–53.7	476,578	46.4	46.3–46.5	1,027,611
Greece	22,343	74.7	74.2–75.2	7,554	25.3	24.8–25.8	29,897
Hungary	73,767	54.2	53.9–54.4	62,441	45.8	45.6–46.1	136,208
Iceland	9,782	44.3	43.7–45.0	12,290	55.7	55.0–56.3	22,072
Ireland	66,978	53.1	52.9–53.4	59,042	46.9	46.6–47.1	126,020
Italy	136,728	61.6	61.4–61.8	85,114	38.4	38.2–38.6	221,842
Latvia	18,343	48.9	48.4–49.4	19,201	51.1	50.6–51.6	37,544
Liechtenstein^a^	70	49.6	41.2–58.1	71	50.4	41.9–58.8	141
Lithuania	26,979	61.7	61.3–62.2	16,741	38.3	37.8–38.7	43,720
Luxembourg	6,502	52.9	52.0–53.8	5,786	47.1	46.2–48.0	12,288
Malta	5,702	64.3	63.3–65.3	3,163	35.7	34.7–36.7	8,865
The Netherlands	179,096	52.9	52.8–53.1	159,284	47.1	46.9–47.2	338,380
Norway	144,679	43.4	43.3–43.6	188,300	56.6	56.4–56.7	332,979
Poland	102,765	64.2	64.0–64.4	57,334	35.8	35.6–36.0	160,099
Portugal	46,499	67.4	67.1–67.8	22,482	32.6	32.2–32.9	68,981
Romania	114,739	67.9	67.7–68.1	54,187	32.1	31.9–32.3	168,926
Slovakia	68,818	50.4	50.2–50.7	67,658	49.6	49.3–49.8	136,476
Slovenia	14,947	58.4	57.8–59.0	10,645	41.6	41.0–42.2	25,592
Spain	269,571	57.5	57.3–57.6	199,353	42.5	42.4–42.7	468,924
Sweden	236,196	47.0	46.8–47.1	266,637	53.0	52.9–53.2	502,833
**Total**	**2,994,557**	**52.9**	**NA**	**2,661,474**	**47.1**	**NA**	**5,656,031**

CI: confidence interval; NA: not applicable.

^a^ Distributions were statistically significant between males and females at the 5% level for all countries except Liechtenstein.

Of note, Denmark and Sweden accounted for 16.2% of all reported cases although their combined populations represented only 3.5% of the average EU/EEA population over the study period. The overall number of cases by disease was driven by three countries accounting for a minimum of 45.1% of all cases (salmonellosis) to a maximum of 68.3% (malaria) (Figure 1). These three countries were most often Germany, France, Italy and Spain. For two diseases, one country accounted for more than 40% of all cases; Germany reported 40.3% of campylobacteriosis cases and France 41.8% of malaria cases. However, there were notable exceptions, such as *C. trachomatis* infection for which Sweden, Denmark and Norway accounted for 57.5% of all cases (Figure 1).

### Proportion of males by disease

The overall proportion of males for the 16 diseases ranged from 35.1% in Estonia to 74.7% in Greece. The overall proportion of males was statistically different from the proportion of females in all countries but Liechtenstein. Eight countries (Estonia, Denmark, Norway, Iceland, Finland, Sweden, Latvia and Liechtenstein) had an overall proportion of males below 50%.

**Figure 2:**
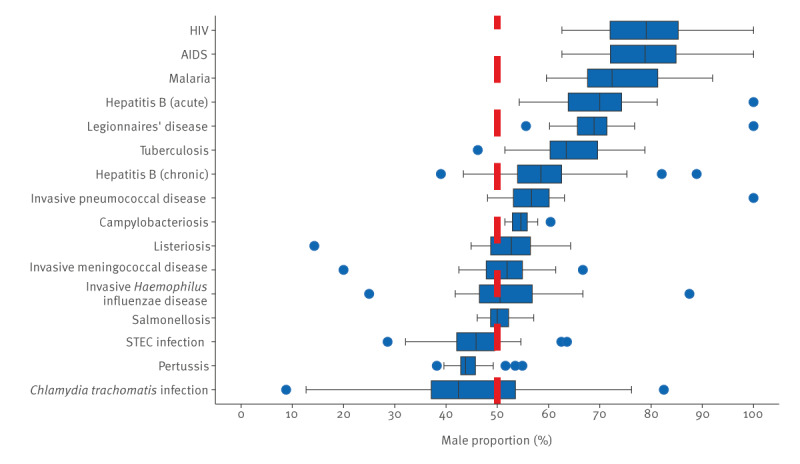
Distribution of proportion of males, by disease, 30 EU/EEA countries, 2012–2021

The proportion of males ranged from 43.1% for *C. trachomatis* infection to 76.6% for HIV (Table 1). These proportions by disease did not differ much from the median of all country proportions by disease, ranging from 42.4% (*C. trachomatis* infection) to 79.1% (HIV) (Figure 2 and Table 3). The proportion of males was statistically different to the proportion of females for all diseases except IHID. For six diseases (campylobacteriosis, acute hepatitis B, Legionnaires’ disease, malaria and HIV and AIDS), all countries had a proportion of males above 50%. A vast majority of countries had also a male proportion above 50% for TB (29/30 countries), IPD (25/27 countries) and chronic hepatitis B (20/24 countries). Conversely, most countries had a male proportion below 50% for pertussis (25/28 countries), STEC infection (21/28 countries) and *C. trachomatis* infection (16/24 countries). For the remining four diseases (salmonellosis, IHID, IMD and listeriosis), the distribution of countries with male proportion above 50% was more even. *Chlamydia trachomatis* infection was the disease with the largest variation in male proportion across countries (median male proportion 42.4%, IQR: 36.1–54.4).

STEC: Shiga toxin-producing *Escherichia coli.*

Boxplots are drawn from interquartile range (IQR) with the boundaries of the whiskers based on 1.5 × IQR value. Outliers are plotted as dots.

**Table 3:** Median, interquartile range and outlier countries with number of cases, for proportion of males, by disease, 30 EU/EEA countries, 2012–2021

Disease	Median	IQR		Outliers country (cases, % males)	
		Q1	Q3	< lower adjacent values	> upper adjacent values
AIDS	78.9	71.7	85.0	None	None
Campylobacteriosis	54.6	52.9	55.9	None	Portugal (4,486, 60.4)
*Chlamydia trachomatis* infection	42.4	36.1	54.4	None	Romania (183, 82.5)
Hepatitis B (acute)	70.0	63.3	74.4	None	Luxembourg (2, 100)
Hepatitis B (chronic)	58.5	53.4	62.8	Denmark (2,268, 39.0)	Luxembourg (216, 88.9) Cyprus (268, 82.1)
HIV	79.1	71.6	85.4	None	None
Invasive *Haemophilus influenzae* disease	50.6	46.4	56.9	Croatia (4, 25.0)	Malta (8, 87.5)
Invasive meningococcal disease	51.9	47.4	55.0	Iceland (10, 20)	Estonia (42, 66.7),
Invasive pneumococcal disease	56.7	52.3	60.1	None	Liechtenstein (1, 100)
Legionnaires’ disease	68.9	65.5	71.4	Iceland (36, 55.6)	Liechtenstein (1, 100)
Listeriosis	52.7	48.7	56.6	Cyprus (7, 14.3)	None
Malaria	72.4	67.0	81.8	None	None
Pertussis	43.8	42.7	45.8	Hungary (110, 38.2)	Cyprus (51, 54.9) Malta (43, 53.5) France (739, 51.6),
Salmonellosis	50.0	48.7	52.2	None	None
STEC infection	45.8	42.0	49.8	Liechtenstein (7, 28.6)	Portugal (11, 63.6) Lithuania (16, 62.5),
Tuberculosis	63.4	60.3	69.6	Liechtenstein (13, 46.2)	None

IQR: interquartile range; Q1: first quartile; Q3: third quartile; STEC: Shiga toxin-producing *Escherichia coli.*

### Proportion of males by disease and age group

**Figure 3:**
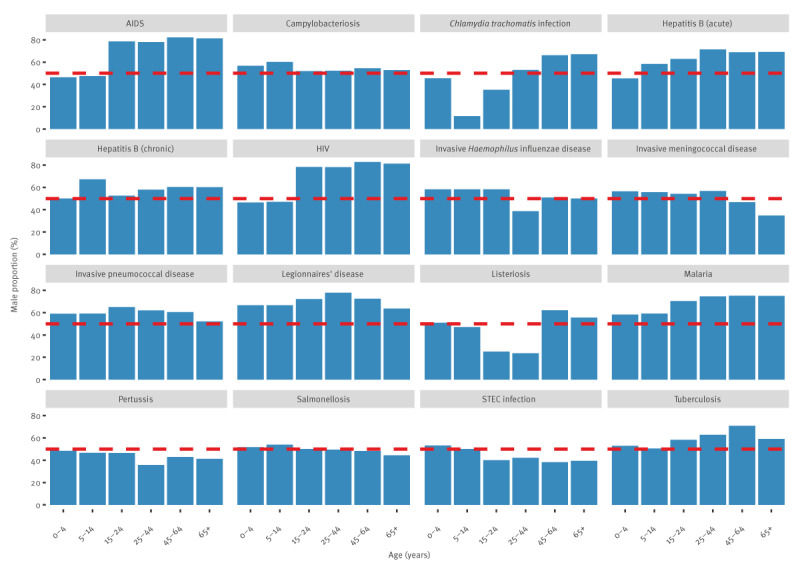
Distribution of proportion of males, by disease and age group, 30 EU/EEA countries, 2012–2021

The dispersion of mean proportion of males by age group varied across diseases (Figure 3). For four diseases (campylobacteriosis, salmonellosis, IPD and malaria), the ratio of the age group with the highest male proportion over the age group with the lowest was less than 1.25. For a second group of five diseases (Legionnaires’ disease, pertussis, STEC infection, and acute and chronic hepatitis B), this ratio was between 1.25 and 1.50. For the remaining seven diseases (TB, IHID, HIV/AIDS, IMD, listeriosis and *C. trachomatis* infection), this ratio was above 1.50. For *C. trachomatis* infection there was an eightfold difference between the male proportion in the 5–14-year-olds (9.8%) and the 65 years and above (78.6%). For listeriosis, the male proportion was 2.7 times higher in the 45–64-year-olds (62.1%) compared with the 25–44-year-olds (23.1%). For HIV and AIDS, the proportion of males increased with age until 15 years and then plateaued around 75%. For IMD, the proportion of males decreased with age after 45 years reaching a lowest in those 65 years and above (33.7%). For IHID, the lowest proportion was observed for the 25–44-year-olds (37.8%).

STEC: Shiga toxin-producing *Escherichia coli.*

Bars show the proportion of males by age group. The 50% threshold is plotted as a red dotted line.

### Outliers

There were no outliers for four diseases (AIDS and HIV, malaria and salmonellosis), i.e. countries with proportion of males beyond upper and lower adjacent values (Table 3 and Figure 2). For the rest of the diseases, most of the outliers were countries with small populations and therefore a small number of cases such as Cyprus, Iceland, Liechtenstein, Luxembourg and Malta. Notable exceptions were the high proportion of males among campylobacteriosis cases reported by Portugal (60.4%) and the low proportion of males among chronic hepatitis B cases reported by Denmark (39.0%).

### Trends

We did not run trend analyses for chronic hepatitis B and pertussis because the countries with no gaps in their timeseries accounted for less than 80% of all reported cases over the study period. Of the 14 remaining diseases, there were significant trends over the study period for eight diseases. Seven diseases (*C. trachomatis* infection, IHID, listeriosis, IPD, TB, STEC infection and campylobacteriosis) had an increasing male proportion over the study period. The proportion of males decreased for malaria. The trends with the highest coefficients were observed for malaria (-0.99, 95% CI: -1.82 to -0.17), IHID (0.41, 95% CI: 0.07 to 0.75), and *C. trachomatis* infection (0.35, 95% CI: 0.25 to 0.46).

## Discussion

Information on biological sex and gender is important for the prevention and control of infectious diseases [1]. The role of biological sex and gender in infectious disease transmission is complex and both context- and disease-specific. In this analysis, in which we only focussed on biological sex, we observed that the proportion of males varied across reported infectious diseases ranging on average from ca 40–45% for pertussis and STEC infection to 75–80% for HIV/AIDS. However, variations between countries were relatively small with low IQR for most diseases. Some sexually transmitted and blood-borne diseases such as HIV/AIDS and hepatitis B had high proportion of males, which is likely to reflect higher risk of infection of males associated with certain transmission modes. Sex between men remained the predominant mode of HIV transmission in the EU/EEA (46% of all new HIV diagnoses in 2021) [14]. Therefore, it is important to target interventions aimed at reducing HIV transmission in men who have sex with men (MSM) with high HIV risk behaviours [15]. In countries with a high proportion of HIV cases reported with unknown transmission mode, male proportion could be a proxy to estimate MSM transmission. For hepatitis B, for which there is a lower proportion of MSM compared with HIV, infection may be associated with both a higher risk of exposure and risk of development of chronic infection in males compared with females [16].

The high proportion of males in malaria cases – which are virtually all imported in the EU/EEA – is likely due to higher exposure in males. An analysis of GeoSentinel data suggested that women were less likely to be infected by vector-borne diseases, such as malaria [17]. In a study among French cases, the sex difference was less pronounced in African individuals visiting friends or relatives compared with white individuals [18]. This may at least partly explain why the male proportion of malaria cases reported by France – a country with a sizeable proportion of the population with African ancestry – was lower than most other countries. Legionnaires’ disease is also known to have a pronounced male bias, possibly due to behavioural differences between sexes, including smoking habits and occupation [7]. Similarly, there are arguments explaining the higher prevalence of TB in males compared with females, including detection bias (e.g. access to care), confounding (e.g. HIV coinfection) and real epidemiological differences [19].

Only two diseases, pertussis and STEC infection, had a male proportion below 50% in the majority of countries. Of the 28 countries reporting pertussis data, 25 had a male proportion below 50% and the remaining three countries had a male proportion below 55%. Notification rates for pertussis are higher in infants compared with older age groups and females are overrepresented in all age groups [20]. The proportion of females was higher above 25 years of age – an age at which the clinical presentation is usually milder than in infants and young children – compared with younger age groups, probably because of different health-seeking behaviour. Recent studies suggested that the source of infection in children was firstly siblings and secondly mothers, excluding the hypothesis that women were more likely to be infected than men because they were more likely to care for sick children. Shiga toxin-producing *Escherichia coli* infections are often linked to the consumption of contaminated food items, mostly beef and fresh produce (fruit and vegetables) [21]. The low proportion of males in STEC infections in adults may be explained by differences in eating habits between sexes. An analysis of STEC outbreaks in the US found that the proportion of females was highest in outbreaks associated with fruits or leafy vegetables whereas sex distribution was even in outbreaks associated with beef and dairy [22].

Large differences in male proportion across age groups can be explained by both risk and testing. Thus, the low proportion of males in listeriosis cases aged 15–44-years is likely to be associated with both a high risk of listeriosis in pregnant women and a high likelihood to be tested when symptomatic because of possible severe pregnancy outcomes [23]. For other diseases such as *C. trachomatis* infection, sex differences are mostly explained by testing policies since chlamydia prevalence in the general population is likely to be similar in both sexes, as suggested by a systematic review [24]. However, chlamydia screening programmes are likely to target sexually active young women. For example, during 2009–2018 in Sweden, 68% of all chlamydia tests were performed on women and 66% of the tested population were in the age group 15–29 years [25]. *Chlamydia trachomatis* infection was also the disease for which male proportion had the highest dispersion across countries, suggesting that very different screening policies were in place.

We only identified a few outliers among countries reporting a large number of cases for a given disease. This was the case of Denmark, where there were 39% males among cases with chronic hepatitis B while the median proportion in all countries was 58.5%. This was probably driven by routine screening of pregnant women in Denmark [26]. Since 2005, all pregnant women have been offered HBV screening in Denmark and participation has been high. Other outliers should be further investigated to determine whether male proportions are associated with overall poor sensitivity of the surveillance system, i.e. its overall ability to detect cases and/or real biases in testing/reporting of male or female cases.

The trends observed in this study are not easily interpretated. Most were modest even if statistically significant. The decreasing proportion of males in malaria cases was partly driven by the low proportion observed in 2021 (55.7%) compared with previous years. It might also reflect a change in travellers’ profile after the release of COVID-19 pandemic-related travel restrictions. The increasing proportion of males in chlamydia infection cases is likely to be associated with an increasing number of cases reported in MSM [27]. The increasing proportion of males in diseases with small sex differences such as listeriosis or IPD remains to be elucidated.

This analysis included more than 5.5 million cases reported by 30 countries over a 10-year-period. The 16 diseases included covered most types of infectious diseases, including food- and waterborne diseases, sexually transmitted infections, vaccine-preventable diseases and vector-borne diseases. Since the late 2000s, ECDC has been coordinating infectious disease surveillance at EU/EEA level with a continuous effort to harmonise surveillance across countries, including the promotion of common EU case definitions. Therefore, surveillance data at EU/EEA level provides a unique opportunity to compare epidemiological patterns and testing or screening policies across countries. Although this study was not able to fully explain the differences observed across countries and diseases, it offers some interesting leads. Based on the likely explanation(s) for these differences, public health professionals should further investigate whether this could apply to their setting to eventually design sex-specific interventions for prevention and control. It also highlighted the importance of documenting some key characteristics of surveillance systems to help interpret surveillance data (Box) [28].

Box **Characteristics of surveillance systems or variables that should be documented to support the interpretation of surveillance data**

- Data sources, especially when only special healthcare services are required to report cases (e.g. maternity hospital).
- The population under surveillance, especially when a specific group is targeted (e.g. prison inmates, who are more likely to be male).
- Case detection policy (e.g. screening of asymptomatic people).
- Variables related to a specific setting of infection (e.g. travel, occupation) or mode of transmission (e.g. sex between men), that may drive a sex difference.
- Information on both sex (male, female, and other) and gender (cisgender, transgender male/female, and other) in surveillance of diseases such as sexually-transmitted infections [29].

This would help identify sex- and gender-based health disparities and eventually design interventions targeting specific groups to prevent and control disease transmission. The way sex and gender are currently captured in TESSy is not fully satisfactory since it mixes biological sex and gender. Although there were only a tiny proportion of cases reported with ‘other sex’, we cannot rule out that some cases were misclassified. However, we believe this is unlikely and therefore would not alter our findings.

## Conclusion

We observed important differences between males and females across infectious disease notifications in EU/EEA countries. However, country variations were relatively small suggesting there may be common drivers for differences across countries. The diseases with the highest male proportions were those for which behaviours play a role in disease transmission, such as HIV. Screening offered to specific populations may explain some differences across countries, such as for *C. trachomatis* infection. It is important to document these characteristics of surveillance systems and collect case information possibly associated with sex/gender differences. In addition, countries can benchmark their male proportion against the EU average to inform public health action such as implementing a screening policy in a specific group.
